# A large-scale screening identifies receptor-like kinases with common features in kinase domains that are potentially related to disease resistance in planta

**DOI:** 10.3389/fpls.2024.1503773

**Published:** 2024-11-13

**Authors:** Yan Huang, Yuan Yuan, Rongqian Yang, Xiangjian Gou, Shiping Dai, Jun Zhou, Jinya Guo, Jinbo Shen, Yanli Lu, Yaxi Liu, Yi Cai

**Affiliations:** ^1^ College of Life Sciences, Sichuan Agricultural University, Ya’an, China; ^2^ Maize Research Institute, Sichuan Agricultural University, Chengdu, China; ^3^ State Key Laboratory of Subtropical Silviculture, Zhejiang A&F University, Hangzhou, China; ^4^ State Key Laboratory of Crop Gene Exploration and Utilization in Southwest China, Sichuan Agricultural University, Chengdu, China

**Keywords:** rRLK, Flg22, PTI signal transduction, plant immunity, Arabidopsis protoplasts

## Abstract

**Introduction:**

The plant genome encodes a plethora of proteins with structural similarity to animal receptor protein kinases, collectively known as receptor-like protein kinases (RLKs), which predominantly localize to the plasma membrane where they activate their kinase domains to convey extracellular signals to the interior of the cell, playing crucial roles in various signaling pathways. Despite the large number of members within the RLK family, to date, only a few have been identified as pattern-recognition receptors (PRRs), leaving many potential RLKs that could play roles in plant immunity undiscovered.

**Methods:**

In this study, a recombinant strategy was initially employed to screen the kinase domains of 133 RLKs in the Arabidopsis genome to determine their involvement in the pathogen-triggered immunity (PTI) pathway. Subsequently, 6 potential immune-related recombinant RLKs (rRLKs) were selected for the creation of transgenic materials and underwent functional characterization analysis. Finally, a sequence analysis was conducted on the kinase domains of these 133 RLKs as well as the known immune RLK receptor kinase domains from other species.

**Results:**

It was found that 24 rRLKs activated the PTI response in Arabidopsis fls2 mutant protoplasts following flg22 treatment. Consistently, when 6 of these rRLKs were individually expressed in fls2 background, they exhibited diverse PTI signal transduction capabilities via different pathways while all retained membrane localization. Intriguingly, sequence analysis revealed multiple conserved amino acid sites within kinase domains of these experimentally identified immune-related RLKs in Arabidopsis. Importantly, these patterns are also preserved in RLKs involved in PTI in other species.

**Discussion:**

This study, on one hand, identifies common features that theoretically can enhance our understanding of immune-related RLKs and facilitate the discovery of novel immune-related RLKs in the future. On the other hand, it provides experimental evidence for the use of recombinant technique to develop diverse rRLKs for molecular breeding, thereby conferring high resistance to plants without compromising their normal growth and development.

## 1 Introduction

As a critical defense mechanism, plants leverage pathogen-associated molecular patterns (PAMPs)-triggered immunity (PTI) to establish a foundational defense against a wide variety of invading pathogens. PTI has recently been found to share downstream responses and connections with effector-triggered immunity (ETI), including ion flux changes, a surge in plant defense hormones and the initiation of a reactive oxygen species (ROS) burst (Yuan et al., 2021). Despite these similarities, PTI and ETI differ in several aspects, such as the pathogen-derived molecules they recognize and some early signaling components (Peng et al., 2017).

The primary elicitors of PTI are PAMPs, which are evolutionarily conserved molecules found in large groups of pathogens and can be composed of proteins, polysaccharides, glycoproteins, or lipopolysaccharides (Zhang and Zhou, 2010). The initiation of PTI hinges on pattern recognition receptors (PRRs), which detect external PAMPs (Zipfel, 2008). Once activated, PRR signals are magnified through mitogen-activated protein kinase (MAPK) cascades, thus initiating downstream signal transduction (Zipfel, 2009). The identified PRRs comprise both receptor-like kinases (RLK) and receptor like proteins (RLPs), where leucine-rich repeats receptor-like kinase (LRR-RLK) stands out as the most abundant class of RLK in plants. LRR-RLKs, characterized by leucine-rich repeat (LRR) domains, a transmembrane (TM) domain, and a functional kinase domain (KD), share structural and functional similarities between plants and animals. These receptors are integral to various signaling pathways, such as CLAVATA (CLV), Brassinosteroids (BR), and XA21, playing pivotal roles in plant development, hormone signaling, and stress responses (Jeong et al., 1999; Müller et al., 2008; Park et al., 2010; Sakaguchi et al., 2010; Clouse, 2011).

Within the genomes of model organisms like *Arabidopsis thaliana* and rice, hundreds of genes encode for LRR-RLKs (Shiu et al., 2004; Gou et al., 2010), but only a subset has been functionally characterized. Due to the specificity of each RLK to certain PAMPs, elucidating their roles presents a considerable challenge. While in-depth studies on RLKs such as Flagellin-sensing 2 (FLS2) and Elongation factor Tu receptor (EFR) are rare (Gómez-Gómez and Boller, 2000; Kunze et al., 2004; Zipfel et al., 2006; Chinchilla et al., 2007; Zeng and He, 2010; Sun et al., 2012; Albert et al., 2013; Mühlenbeck et al., 2024), it has been discerned that the signaling cascades triggered by PRRs exhibit considerable overlap (Mukhtar et al., 2011). This implies a potentially conserved mechanism of action among various RLKs following pathogen recognition. The modular nature of RLKs is evidenced by the successful swapping of extracellular domains (responsible for PAMP detection) and kinase domains (critical for signal transduction) among different RLKs without impairing their function (Albert et al., 2010; Brutus et al., 2010; Mueller et al., 2012; Albert et al., 2013; Ma et al., 2022; Lu et al., 2023). This modularity facilitates the functional analysis of less characterized RLKs, as well-established domains from known RLKs can be used to investigate the functions of unknown counterparts. This approach can significantly streamline the unraveling of the intricate array of RLKs and their roles in plant immunity and overall biology.

Building on these foundations, our investigation leveraged a transient protoplast expression system to screen recombinant RLKs (designated as rRLKs) engineered by fusion of the extracellular and transmembrane domains from Arabidopsis FLS2 (FLS2 NT) with the kinase domains (KDs) from an array of 133 candidate RLKs. Our preliminary investigation involved assessing their role in PTI by measuring their efficacy in activating the disease resistance reporter, *FRK1::Luciferase* (*FRK1::LUC*). Subsequently, plants genetically engineered to express the screened rRLKs were evaluated for disease resistance capabilities through physiological and biochemical methods. Accompanied by sequence analysis, we have summarized the molecular characteristics associated with RLK-mediated disease resistance, providing valuable insights for RLK research across different species.

## 2 Materials and methods

### 2.1 Plant growth conditions and candidate RLKs selection

Generally, *A. thaliana* plants used in this study were grown in a phytotron under exposure to an 8/16 h light/dark cycle, at a temperature between 22°C and 24°C, relative humidity of 45% and illumination of 5500 LX. For the protoplast preparation and detection of callose deposition, all the plants are cultivated under short day chamber, with 12 h light/12 h dark, temperature 22°C, humidity 40%, and light 5500 LX.

133 *A. thaliana LRR-RLKs* were selected as experimental genes from the Ensembl Plants database (Howe et al., 2020) and protein domain prediction tool SMART (NORMAL mode) (Letunic et al., 2021) (**Supplementary Table S1**).

### 2.2 Plasmid constructions and selection of homozygous transgenic lines

For transient expression experiment in Arabidopsis protoplasts, 134 *RLK KD* fragments were amplified using wild-type cDNA as templet, which carrying 15 bp homologous sequences of both ends of the vectors at each end by specific gene primers. After digesting the vectors HBT95-*FLS2 NT-GFP* (previously constructed in the lab) with *Nco*I and *Kpn*I, above amplified fragments were cloned into the linearized vectors by fusion cloning method, respectively, to generate 133 HBT95-*FLS2 NT*-*RLK KD-GFP* plasmids plus the positive control plasmid HBT95-*FLS2 NT*-*FLS2 KD-GFP*. For stable transgenic plants construction, the coding sequences of the 6 selected RLK KD domains were obtained by digesting HBT95-*FLS2 NT*-*RLK KD-GFP* with *Bam*HI and *Pst*I, and cloned into pCamBia1305-*FLS2 NT-GFP* (previously constructed in the lab) to generate pCamBia1305*-FLS2 NT-RLK KD-GFP*. These constructs were then transferred into *Agrobacterium tumefaciens* strain GV3101 by heat shock method and subsequently transformed into *fls2* mutant plants by floral dipping (Clough and Bent, 1998).

For selection of homozygous transgenic lines, T_1_ generation transgenic plants that exhibit hygromycin resistance are harvested individually to obtain seeds for the T_2_ generation. Approximately 40 seeds from each line are then planted and their segregation ratios on resistance plates are used to determine whether they consist of single-copy insertions (with 3/4 of the plants being resistant). From the T_2_ lines judged to be single-copy insertions, 12 resistant seedlings are randomly chosen (of which theoretically 1/3 should be homozygous lines) for transplantation and individual harvesting to obtain T_3_ generation seeds. The T_2_ plants corresponding to T_3_ lines where all seeds survive on resistance plates are considered to be homozygous lines. Two independent homozygous transgenic lines are then selected for further characterization and analysis.

### 2.3 Protoplast preparation and transfection

Method for extracting mesophyll protoplasts (Yoo et al., 2007) from Arabidopsis *fls2* mutants is described below. Briefly, 4-week-old *fls2* leaves were cut into 0.5-1 mm filaments and quickly immersed in an enzymatic hydrolysis solution (0.4% macerozyme R10 and 1.5% cellulose Onozuka R10). Under the same conditions of plant culture without light, the leaves were hydrolyzed for 4 h before an equal volume of W5 buffer (2 mM MES, pH 5.7; 154 mM NaCl; 125 mM CaCl_2_; 5 mM KCl) was added, which was subsequently filtered through 350-µm nylon mesh and centrifuged at 667 rpm for 2 min. The supernatant was removed via vacuum suction and the protoplasmic cells were re-suspended by suitable W5 before placed on ice for 30 min to deposit the protoplasmic cells. After removing W5, the concentration of protoplasts was adjusted by MMG buffer (4 mM MES, pH 5.7; 15 mM MgCl_2_; 0.4 M mannitol) to 2×10^5^ cells/mL to facilitate subsequent transformation experiment.

Polyethylene glycol (PEG)-mediated transfection (Yoo et al., 2007) was performed to transfer total 10 µL plasmids (*35S::FLS2 NT-RLK KD-GFP*+ *FRK1::LUC (* Asai et al., 2002)+ *UBQ10::GUS* (Sheen, 2001)) into *A. thaliana* protoplasts before incubating (at 24°C and 2000 LX) in W5 buffer for 11-12 h, and then treated with 1 µM flg22 for another 12 h.

### 2.4 Luciferase and β−glucuronidase assays

After treatment with flg22, the above protoplasts were harvested by centrifuging at 667 rpm for 2 min at 4°C for luciferase and β-glucuronidase (GUS) activity test (Popov et al., 2016), which method was described as follows. Firstly, 50 µL cell lysate was added to the collected protoplasts and a cell crusher was used to assist in full lysis. Then, 5 µL of the above protoplast lysate was added into a 96-well plate mixing with 100 μL luciferin before the luciferase activity was tested with a Perkin Elmer VICTOR X3 (Waltham, Massachusetts, USA). On the other hand, 2 µL protoplast lysate was added into a 96-well plate mixing with 25 µL MUG buffer (10 mM Tris-HCl, pH 8; 2 mM MgCl_2_; 1 mM 4-methyl-umbelliferyl-β-D-glucuronide), which was then incubated at 37°C for 30 min before adding 225 μL 0.2 M Na_2_CO_3_ to terminate reaction. Finally, the GUS activity was measured by the Perkin Elmer VICTOR X3.

### 2.5 Subcellular localization

6 recombinant *35S::FLS2 NT-RLK KD-GFP* transient expression vectors were individually co-transfected with *35S::SCAMP1-RFP* expression vector into *fls2* protoplasts, and the localization of fluorescent proteins was observed by a confocal microscope (OLYMPUS FV1200, Japan) after 12 hours of cultivation. Confocal images of 6 rRLK-GFP fluorescence in root cells of plate-grown *fls2* transgenic plants were obtained by a confocal microscope (OLYMPUS FV1200, Japan). GFP signal was detected at 488 nm excitation (500-540 nm emission, HyD3 detector).

### 2.6 Callose deposition assay

Leaves from 4-week-old plant were infiltrated with 1 μM flg22 or ddH_2_O for 18 h, respectively, and soaked in the six-well plate with 4 mL fixative (ethanol∶ acetic acid = 3∶ 1) for 1 h. After replacing the fixative with 50% ethanol, the plate was incubated at 65°C for 15 mins for decolorization. Then, the ethanol was taken away and the samples were treated with 150 mM K_2_HPO_4_ (pH 9.5) solution containing 0.01% Methyl Blue for 1 h in the dark. Finally, glycerol was added to preserve the samples after blotting out the staining solution, and pictures were taken using fluorescence microscope for callose deposition analysis.

### 2.7 Gene expression analysis

About 80 mg plant tissue was collected from 14-day-old seedlings grown on 1/2 MS medium after 1 μM flg22 or ddH_2_O treatment. RNA was extracted using the EASY spin Plus Plant RNA kit (TaKaRa), which then reverse transcribed by HiScript^®^ III RT SuperMix for qPCR (+gDNA wiper) (Vazyme) to obtain cDNA. cDNA samples were initially normalized with *ACTIN2* by real-time PCR using the ChamQ Universal SYBR qPCR Master Mix kit (Vazyme). The primers used for amplification of *ACTIN2*, *FRK1*, *PR1* and *WRKY33* were as follows. *ACTIN2*, 5’-CTTGTTCCAGCCCTCGTTTG-3’ and 5’-CAGCGATACCTGAGAACATAGTG G-3’; *FRK1*, 5’-TCTGAAGAATCAGC TCAAGGC-3’ and 5’-TGTTGGCTTCACATCTCTGTG-3’; *PR1*, 5’- ACGCAGAAC AACTAAGAGGCAAC-3’ and 5’- AGCCTT CTCGCTAACCCACAT-3’; *WRKY33*, 5’- GCTGCTATTGCTGGTCACTCC-3’ and 5’-T GCGTTTGAAGGTTGCTGTT-3’.

### 2.8 Pathogen infection

For virulent *Pseudomonas syringae* pv. *tomato* (*P.s.t.*) strain DC3000 infection, bacterial cultures diluted to OD_600_ = 5×10^-4^ with 10 mM MgCl_2_ were used to infiltrate leaves of around 4-week soil-grown plants. Leaf disks from the infected areas were taken at 0 d and 3 d after infiltration to quantify the bacterial colony-forming units (cfu) on LB plates with Kanamycin and Rifampicin antibiotic selections.

### 2.9 Protein extraction and detection of phosphorylation of MAPKs

70 mg leaf tissue from 15-day-old 1/2 MS medium-grown plants was collected after 1 μM flg22 treatment for 5 min and ground into powder by liquid nitrogen. The samples were homogenized in extraction buffer (10% 1 M Tris-HCl, pH8; 0.1% SDS; 2% β-mercaptoethanol; 0.1% 1 M DTT; 1% PMSF), and the supernatant of each sample was obtained by centrifuging at max speed for 5 min. After adding 4× SDS loading buffer (60 mM Tris-Cl, pH6.8; 2% SDS; 5% β-mercaptoethanol; 10% glycerol; 0.01% bromophenol blue) to a final concentration of 1×, the samples were boiled at 95°C for 5 min and stored at -20°C.

The above protein samples were separated by 10% SDS-PAGE. For MAPK phosphorylation detection, western blotting was carried out using an antibody specific for p42/44-MAPK (Cell Signalling Technology, Danvers, MA, USA) to determine whether the phosphorylation of MPK3 and MPK6 was affected.

### 2.10 Identification of immune-related amino acid sites in RLKs’ KD

For the sake of discovering some common features of kinase domain in the immune-related rRLKs, multiple sequence alignment was executed by Clustal Omega (v: 1.2.4) (Sievers and Higgins, 2018; Madeira et al., 2024).

Furthermore, to verify whether these crucial amino acids were also conserved across species, 13 LRR-RLKs in other species that have been confirmed to be immune-related were screened from NCBI database, and their KD regions were predicted by SMART (Letunic et al., 2021) (**Supplementary Table S2**). The kinase domain of FLS2 in Arabidopsis were multiply aligned with the kinase domain of the 13 RLKs in other species (denoted as KD-F-O below, that is KD-FLS2-Other species) using Clustal Omega (Sievers and Higgins, 2018).

Specifically, we focused on the alignment of three groups (immune-positive group “KD-p” which includes 109 KDs of RLKs identified from our protoplast screening that do not activate PTI, immune-negative group “KD-n” encompassing 24 KDs from RLKs that were identified to activate PTI to varying degrees in the protoplast screening and other species group “KD-F-O” consisting of 16 published KDs of RLKs from other species known to be involved in immunity), and compared their conserved amino acid sites based on Clustal Omega and its visual tool Jalview (v: 2.11.3.3) (Waterhouse et al., 2009): (I) Initially, highly conserved residues within the KD-p group were identified, serving as potential characteristic sites of immune-associated RLKs. (II) Subsequently, these candidate residues were cross-referenced against highly conserved residues within the KD-n group to eliminate common fundamental characteristics present in KDs across all LRR-RLKs. This step was crucial to pinpoint sites with a high likelihood of being related to immune functions. (III) Finally, these putative immune-related sites were compared with corresponding sites in the KD-F-O group to assess whether these key sites exhibited conserved traits across different species.

## 3 Results

### 3.1 Multiple rLRR-RLKs can effectively transmit PTI signals and activate the downstream reporter gene *FRK1* in Arabidopsis protoplasts, achieving activation levels comparable to or stronger than that of WT FLS2

**Figure 1:**
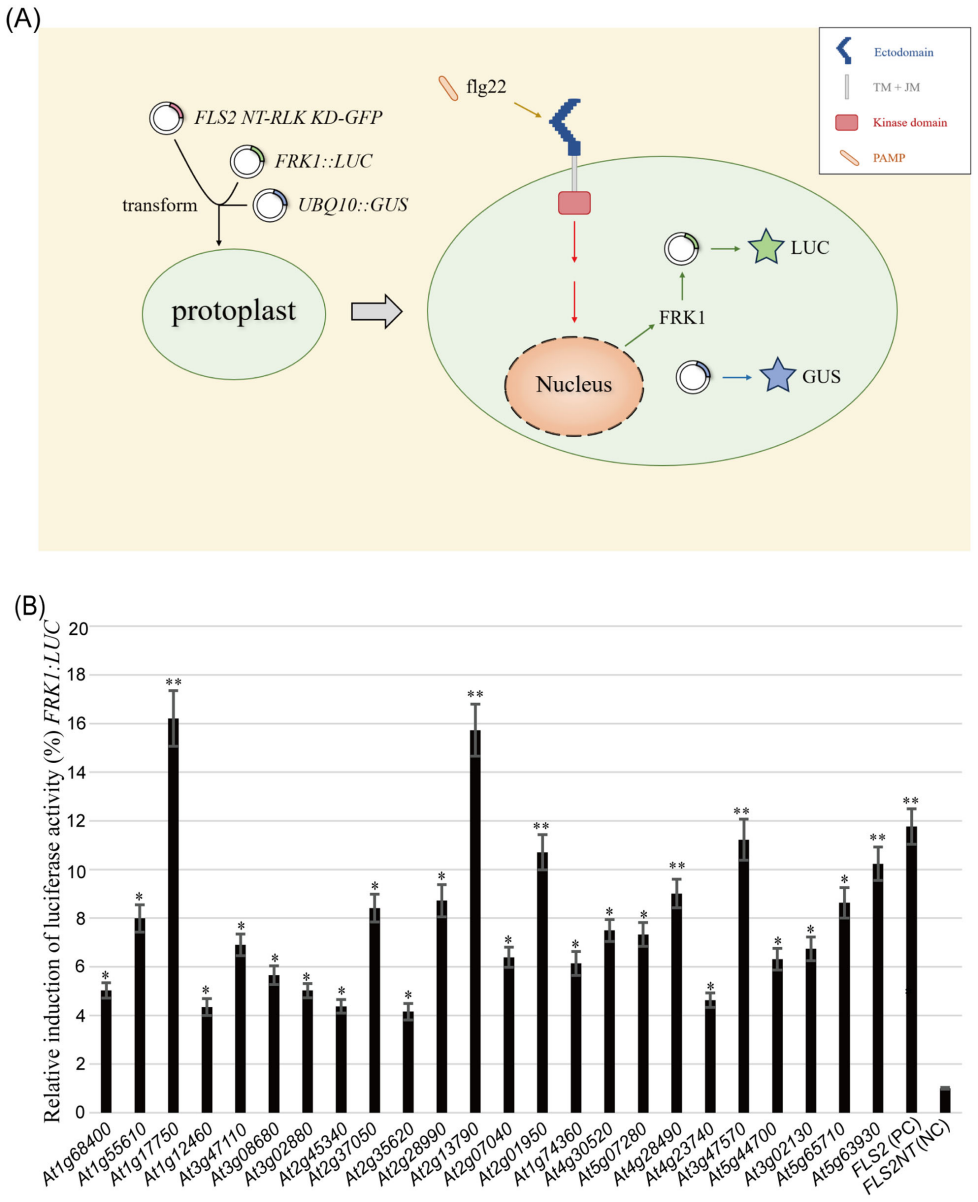
Screening for potential rRLKs involved in PTI immune response using the Arabidopsis protoplasts transient expression system. **(A)** Screening process. **(B)** Co-transformation of 24 HBT-*FLS2 NT-RLK KD-GFP*, HBT-*FLS2 NT-FLS2 KD-GFP* (Positive Control, PC) or HBT-*FLS2 NT-GFP* (Negative Control, NC) with (*FRK1::LUC* + *UBQ10::GUS*). the *FRK1* relative expression after flg22 induction was determined by dividing the experimental group LUC/GUS by the negative control group LUC/GUS. Bars represent means of 5 replicates ± SD. The experiments were repeated 3 times with similar results. * indicates significant difference at P<0.05; ** indicates significant difference at P<0.01.

Utilizing a recombinant RLK approach, 133 HBT-*FLS2 NT-RLK KD-GFP* transient expression vectors (**Supplementary Table S1**) were successfully constructed. By means of the Arabidopsis protoplast transient expression system, whether these RLK KDs participated in plant immune signal transduction was screened and identified (**Figure 1A**). Among them, a total of 24 RLK kinase domains after flg22 treatment could proficiently mediate PTI pathway signal transduction and effectively trigger the activation of the downstream reporter *FRK1*, with HBT-*FLS2 NT-FLS2 KD-GFP* serving as the positive control and HBT-*FLS2 NT-GFP* as the negative control (**Supplementary Figure S1**, **Figure 1B**). Of the 24 RLKs, the functions of 16 (Wang et al., 2001; Caño-Delgado et al., 2004; Huffaker and Ryan, 2007; Tsuwamoto et al., 2008; Kinoshita et al., 2010; Chang et al., 2013; Xiao et al., 2015; de Oliveira et al., 2016; Huang et al., 2016; Patharkar et al., 2017; Wang et al., 2017; Hu et al., 2018; Isner et al., 2018; He et al., 2021; Huang et al., 2023; Chang et al., 2024) have been reported to be either related to immunity or development.

Notably, 6 RLK KDs derived from At1g17750 (PEPR2) (Huffaker and Ryan, 2007), At2g13790 (SERK4) (de Oliveira et al., 2016), At2g01950 (BRL2) (Wang et al., 2001), At4g28490 (HAESA) (Patharkar et al., 2017), At3g47570 and At5g63930 (PSYR3) (Ogawa-Ohnishi et al., 2022) demonstrated comparable or even superior functionality to the WT FLS2 KD in activating the PTI signaling pathway (**Figure 1B**). Although reports have identified the homologous gene *HaOr7* in sunflower, which encodes a RLK protein that confers resistance to *Orobanche cumana* race F (Duriez et al., 2019), there have been no reports to date regarding whether *AT3G47570* is involved in plant immunity in Arabidopsis.

### 3.2 rLRR-RLKs tested in Arabidopsis protoplasts were located on the plasma membrane when expressed in *fls2* background

**Figure 2:**
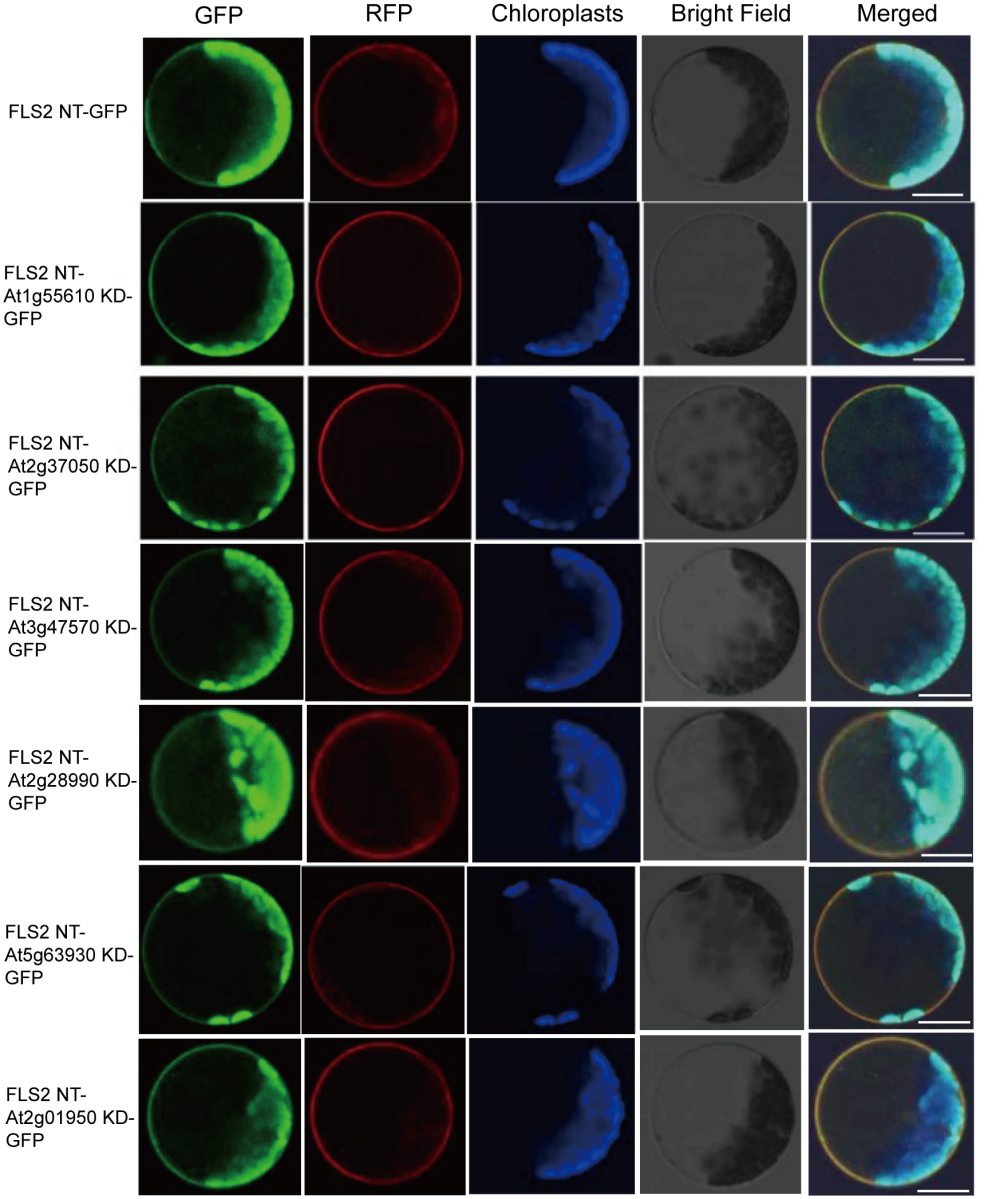
Subcellular localization of rRLK-GFP proteins in protoplasts. Confocal images of 6 rRLK-GFP fluorescence in protoplasts when individually expressed with membrane marker coding gene *35S::SCAMP1-RFP*. Bars = 20 μm.

In order to ascertain whether the above identified RLKs do function in immune response, 6 rRLKs-GFP including FLS2 NT-At1g55610 (BRL1) (Caño-Delgado et al., 2004) KD-GFP, FLS2 NT-At2g37050 (SIMP1) (He et al., 2021) KD-GFP, FLS2 NT-At3g47570 KD-GFP, FLS2 NT-At2g28990 KD-GFP, FLS2 NT-At5g63930 KD-GFP, FLS2 NT-At2g01950 KD-GFP) that can respond to PTI elicitor signals and activate downstream immune responses in protoplasts to varying degrees were selected to further construct *fls2* OE *rRLK-GFP* transgenic plants in this study. Firstly, as plant signal receptors, these rRLKs can effectively transmit signals from the extracellular environment to the intracellular milieu only if they are correctly localized within the cell. Therefore, we initially examined the GFP fluorescence localization of the 6 rRLKs-GFP both in protoplasts and in each selected transgenic homozygous line. As demonstrated in **Figure 2**, all the tested rRLK fusion proteins co-localized with the plasma membrane marker SCAMP1-RFP in protoplasts. Moreover, they were normally expressed in the *fls2* background and appropriately localized to the plasma membrane as anticipated (**Supplementary Figure S2**).

### 3.3 *fls2* expressing pre-screened rLRR-RLKs encoding genes had no negative effects on its growth and development

**Figure 3:**
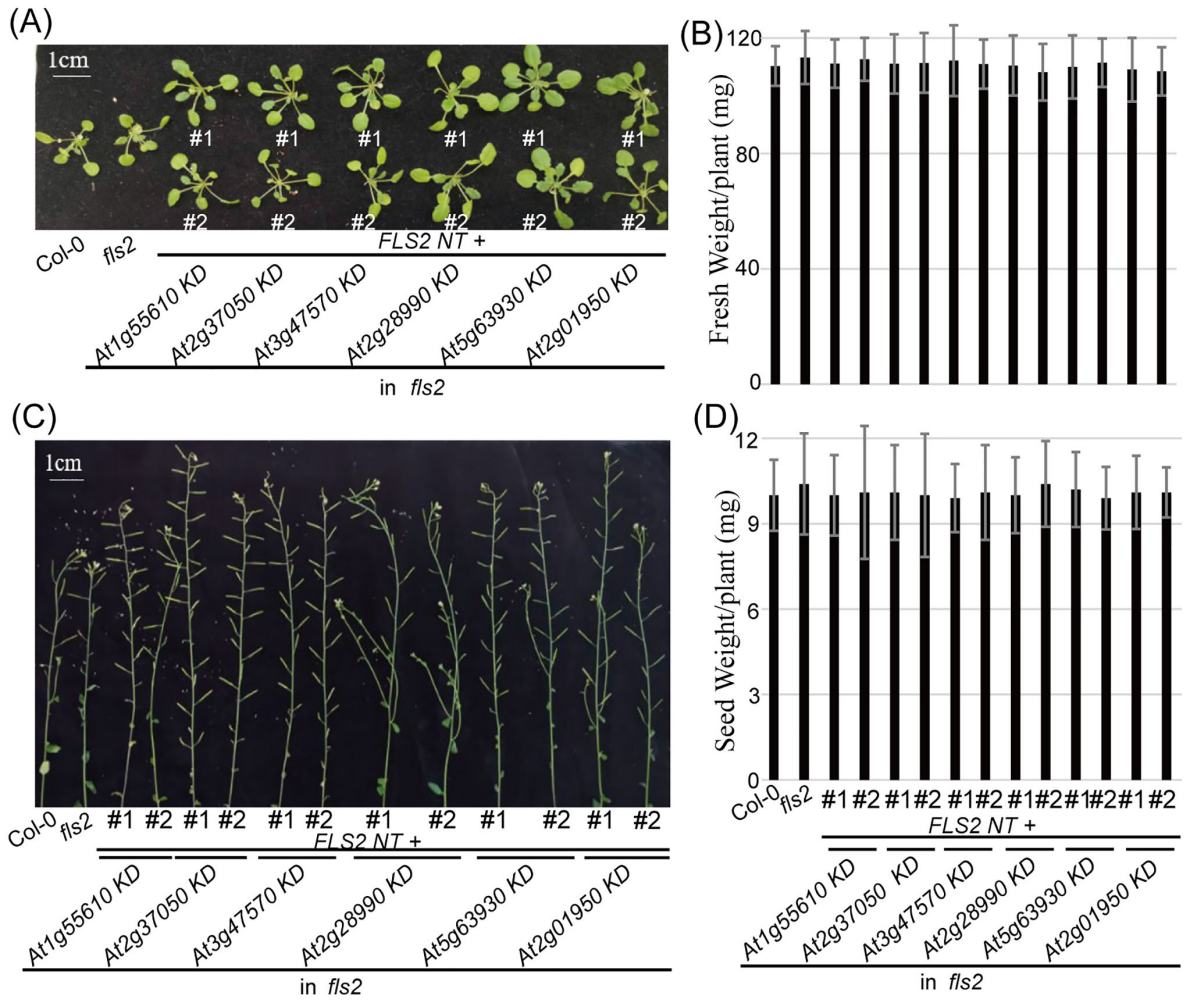
Morphology of 6 *rRLK-GFP* transgenic plants in the *fls2* mutant background. **(A)** Morphological phenotype of vegetative growth stage. Bar = 1 cm. **(B)** Fresh weight of 4-week-old plants of the indicated genotypes. Bars represent means of 12 replicates ± SD. The experiments were repeated 3 times with similar results. **(C)** Morphological phenotype of reproductive growth stage. Bar = 1 cm. **(D)** Seed weight from 10-week-old plants of the indicated genotypes. Bars represent means of 12 replicates ± SD. The experiments were repeated 3 times with similar results.

In our examination of the transgenic plants expressing the pre-screened rLRR-RLKs, it was imperative to ensure that the presence of these recombinant proteins did not impinge on normal plant functions. Consistent with the elicitor induction required for the occurrence of PTI, the morphological evaluation showed that these homozygous transgenic lines revealed no notable deviations from the Col-0 and *fls2* control groups throughout their entire life cycle (**Figure 3**). Correct localization of these rRLKs confirmed their potential to function as intended, and crucially, their expression did not automatically provoke an autoimmunity response in the plants. Therefore, we concluded that the engineered PTI receptors did not negatively impact plant vitality or reproductive processes.

### 3.4 *fls2* expressing pre-screened rLRR-RLKs encoding genes exhibited different PTI signal transduction abilities and their signal transduction pathways were diverse

**Figure 4:**
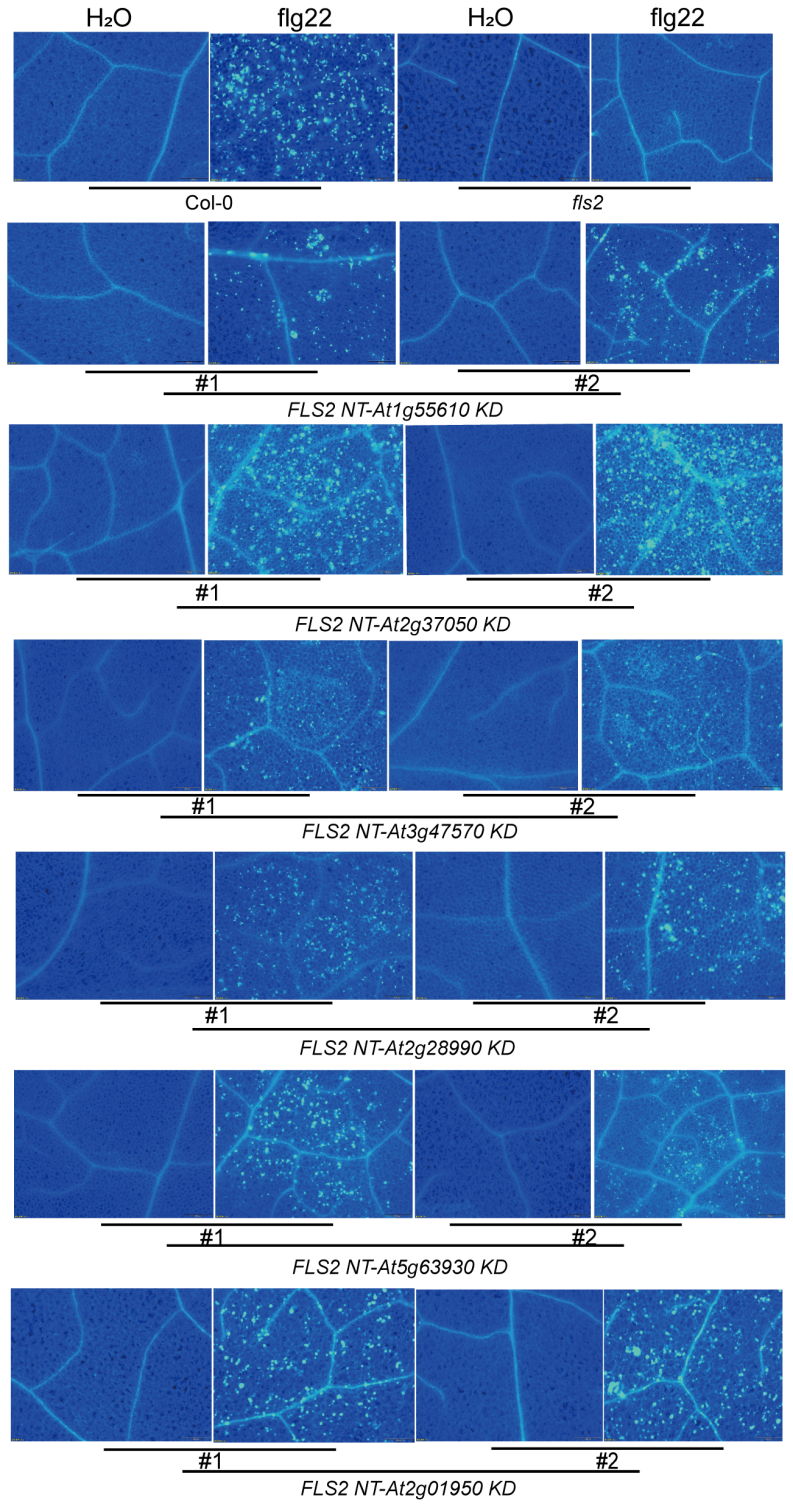
Accumulation of callose deposition in tested plants induced by H_2_O and flg22. Using Col-0 and *fls2* as control, callose deposition in leaves of transgenic plants individually expressing 6 *rRLK-GFP* after 1 μM flg22 or ddH_2_O treatment was observed by fluorescence microscope.

**Figure 5:**
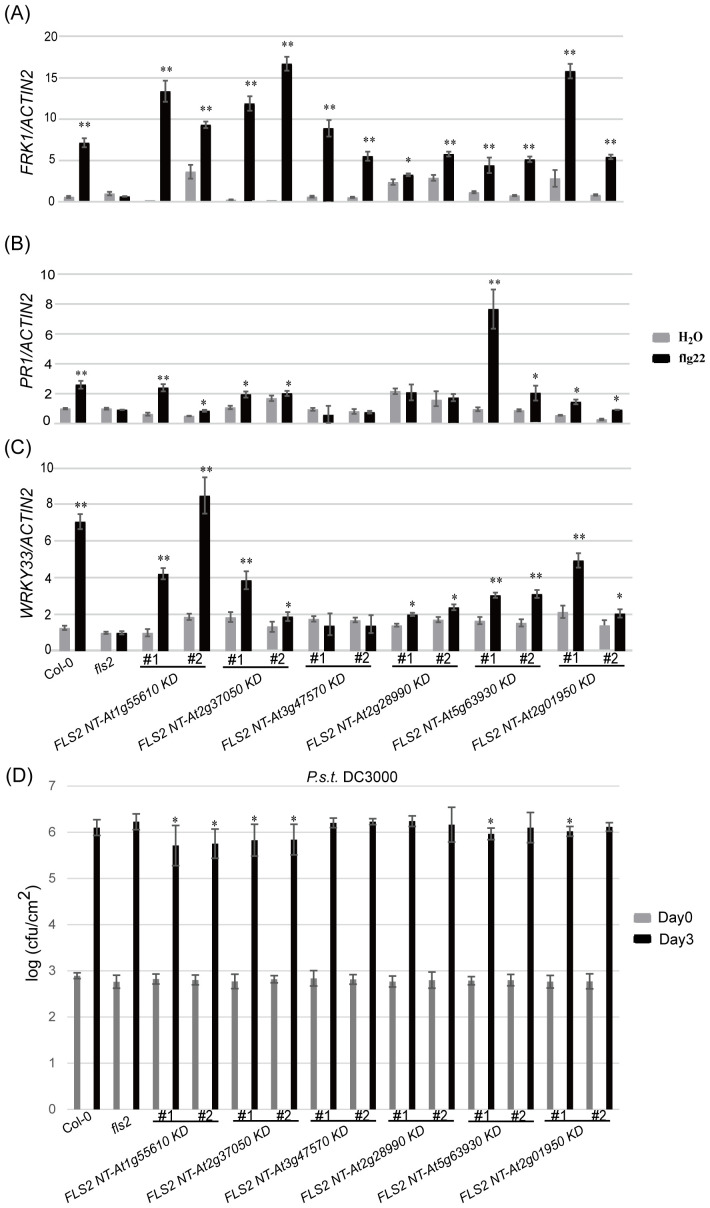
Analysis of resistance characteristics of each tested plant. **(A–C)** represents the relative expression levels of defense marker gene *FRK1* **(A)**, *PR1* **(B)** and *WRKY33* **(C)** in the background of each tested plant induced by H_2_O and flg22. Bars represent means of three replicates ± SD; * indicates significant difference at P<0.05; ** indicates significant difference at P<0.01. **(D)** Bacterial growth of *P.s.t.* DC3000 on each tested plant. Data are means (± SD), n=5 (five independent samples from one time assay); * indicates significant difference at P<0.05. The experiments were repeated 3 times with similar results.

Continuing our analysis, we delved into the PTI signaling capacity of these rRLKs and discovered diversity in their subsequent signaling pathways. Through an array of resistance characterization assays including callose deposition, defense marker gene expression and resistance against *P.s.t.* DC3000, we sought to delineate the impact of these 6 rRLKs on the PTI pathway in Arabidopsis. Unlike *fls2* with no response to the induction of flg22, the level of callose deposition in the transgenic background recovered to varying degrees after flg22 treatment, and some lines almost recovered to levels similar to or even higher than that of Col-0 (**Figure 4**). Consistent with the transient screening results, all *rRLK* transgenic lines could respond to the induction of flg22 and produce high levels of *FRK1* expression (**Figure 5A**), while they had discrepant expression levels of *PR1* or *WRKY33* under flg22 induction (**Figures 5B, C**). Notably, the KD kinase domains from At1g55610, At2g37050, and At2g01950 or from At5g63930 appear to facilitate more robust signal transduction than WT FLS2 in the *FRK1* or *PR1*-dependent pathway, respectively, ultimately conferring heightened resistance against the virulent bacterial pathogen *P.s.t.* DC3000 in plants (**Figure 5D**).

**Figure 6:**
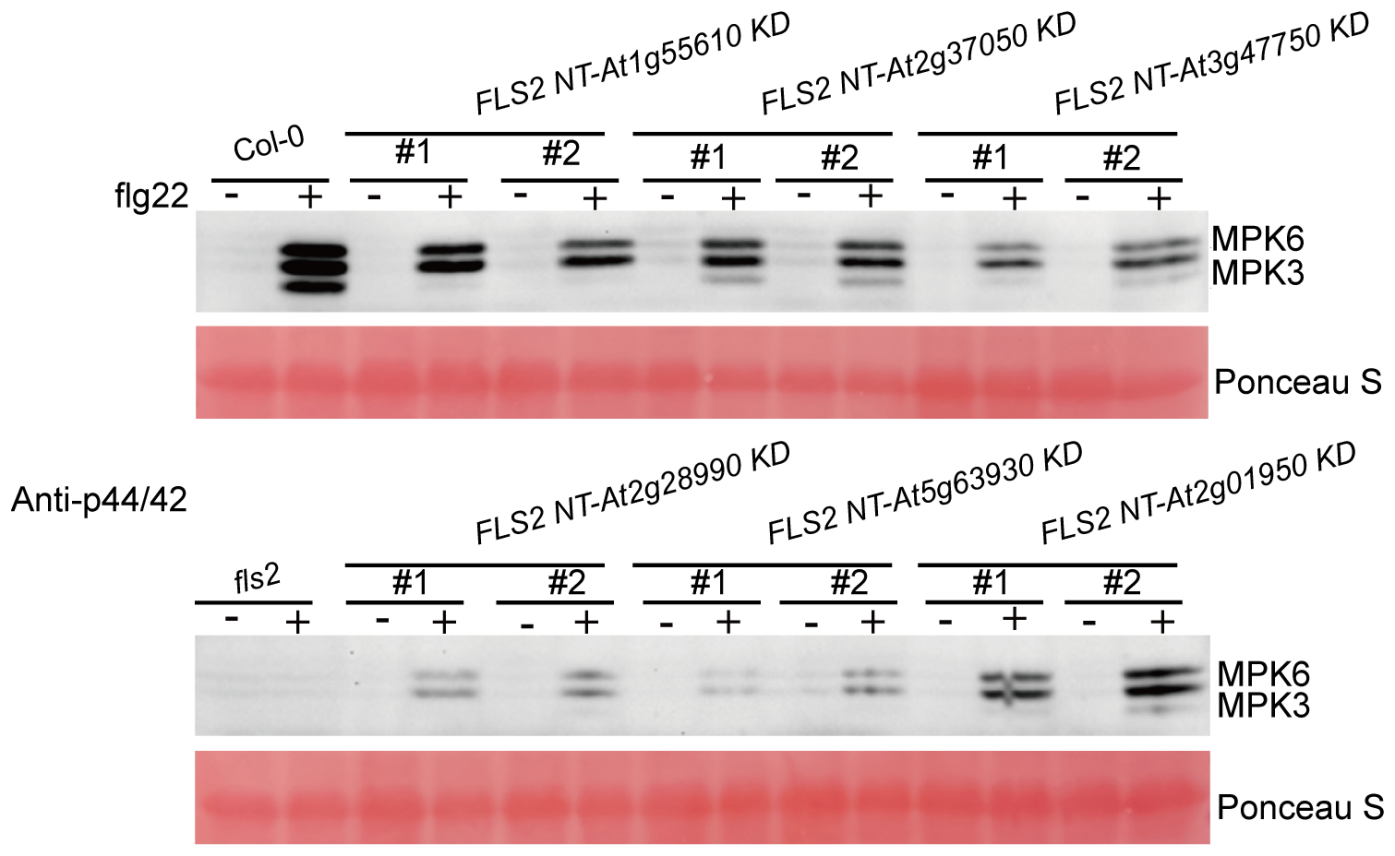
Detection of MAPKs phosphorylation induced by H_2_O and flg22 in the indicated genotypes. MAPKs phosphorylation induced by H_2_O and flg22 in the indicated genotypes was examined by Western blot with a specific anti-p44/42 antibody (Cell Signaling Technology). Rubisco levels from Ponceau S staining served as an internal loading control. The experiments were repeated 3 times with similar results.

Findings demonstrate that all 6 kinase domains (from At1g55610, At2g37050, At3g47570, At2g28990, At5g63930, At2g01950), when fused with FLS2 NT, could mediate transmission of the PTI elicitor signal, triggering immune-related physiological changes in transgenic plants. These alterations, however, differed among the transgenic lines in terms of callose deposition and flg22-induced expression of defense genes. This suggests that, while the rRLKs may utilize the same FLS2 NT for signal reception, their downstream signaling pathways diverge, potentially orchestrating differing defense responses via distinct routes. Western blot analyses were performed to tentatively examine the underlying mechanisms of these enhanced disease-resistant signals in rRLKs (**Figure 6**). Results revealed that flg22 induction led to varied enhancements in MPK3 and MPK6 phosphorylation, indicating a MAPK cascade-dependent pathway in mediating plant immune responses across these kinase domains.

### 3.5 rLRR-RLKs involved in PTI immune response share conserved amino acid sites through bioinformatics analysis

**Figure 7:**
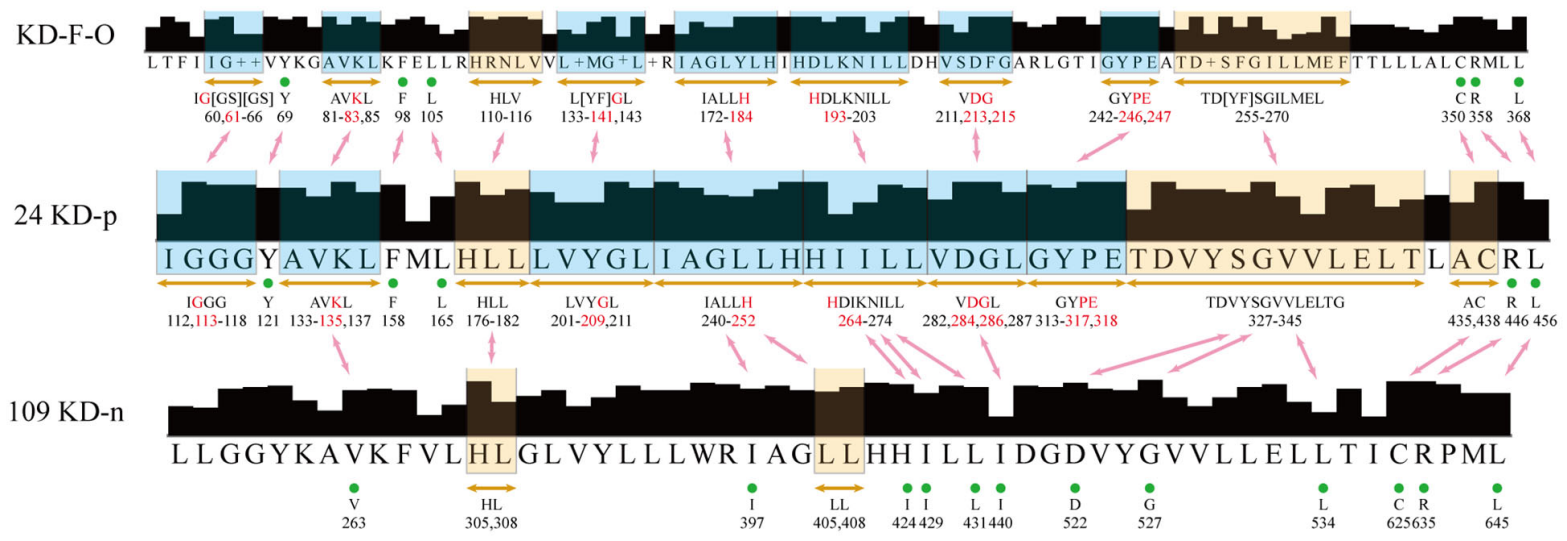
Alignment result of the three groups. The boxes indicate a series of approximately consecutive conserved amino acid sites, while the green dots indicate individual conserved sites. The red characters are the most potentially immune-related sites, which have 100% conservation in 24 KD-p, but low conservation in 109 KD-n. The blue boxes are the regions with the most potentially immune-related sites. The red arrows show the correspondence between two groups.

Building upon the insights gleaned from the clustering of immune-related rRLKs, we integrate bioinformatics analyses to spotlight the shared conserved amino acid residues critical to the KD function in plant immunity. The alignments showcase 57 highly conserved sites across 24 KDs that are positively linked to immune signaling (referred to as KD-p). Moreover, these 57 sites encompass 16 sites that are also deeply conserved within a broader set of 109 KDs that do not activate PTI (labeled as KD-n), indicating their foundational role in the structural integrity and function of LRR-RLKs in general. Remarkably, a subset of 9 sites out of these 57, a group marked by absolute conservation within KD-p but not within KD-n, emerges as particularly promising candidates for immune-specific functionality. These sites are also found to be conserved across an array of LRR-RLKs in different species (known as KD-FLS2-Other or KD-F-O), implicating them as critical determinants in the activation of immune signaling across the plant kingdom (**Figure 7**, **Supplementary Table S3**).

This assessment, drawing connections between the protoplast pre-screening experiment and broad phylogenetic data, reinforces the notion that key conserved sites within rLRR-RLKs are not just vestiges of protein architecture, but are actively engaged in sensing and defending against external biotic stress. By highlighting these sites’ preservations in related species’ RLKs—spanning EFR in Brassica (Zhang et al., 2018), FLS2 variants in grapevine (Trdá et al., 2014), tomato (Robatzek et al., 2007; Hind et al., 2016), and other Solanaceae members (Wang et al., 2016; Wang et al., 2018; Xu et al., 2018), and beyond (Soderlund et al., 2009) (**Supplementary Table S2**), the analysis underscores the evolutionary importance of these domains in plant immunity.

## 4 Discussion

### 4.1 Recombination strategy can advance the understanding of unknown RLK functions

For a long time, it was believed that plants lacked an immune system. However, with the development of botany and in-depth research into the molecular mechanisms underlying plant disease resistance, it has become clear that, similar to animals, plants have evolved a unique multi-layered innate immune system to respond to pathogen infections in their environment (Jones and Dangl, 2006; Spoel and Dong, 2012; Cheng et al., 2019). The most fundamental component of the plant immune response is pattern recognition, which includes the specific recognition of the conserved pathogen PAMPs by PRRs on the surface of plant cell membrane, and the detection of pathogen-secreted avirulence proteins by intracellular immune receptor-resistance (R) proteins (Boller and Felix, 2009; Lolle et al., 2020). Among these components, PTI serves as the first line of defense for plants against pathogens. The key to triggering this response lies in the recognition of specific PAMPs by PRRs. To date, multiple PRRs have been identified in plants using molecular biology, proteomics, and cell biology methods. Most of these PRRs belong to the LRR-RLK class and play crucial roles in various signal transduction pathways. These PRRs can specifically recognize corresponding ligands to activate the intracellular kinase domains, thereby transmitting extracellular signals into the cell through either self-phosphorylation or co-phosphorylation with their co-receptors, and then dynamically regulate plant growth, development and immune response (Clark et al., 1997; Gómez-Gómez and Boller, 2000; Fisher and Turner, 2007; Robatzek et al., 2007; Kinoshita et al., 2010; Furukawa et al., 2014; Sun et al., 2017). Despite the large number of RLKs encoded by the genomes of various species, the functions of only a small fraction of them have been elucidated, primarily because many of their corresponding ligands remain unknown. Fortunately, early studies revealed that recombinant RLK (rRLK) can still successfully mediate the plant immune responses when the extracellular domain and transmembrane domain (NT) of one RLK are recombined with the kinase domain (KD) of another RLK (Albert et al., 2010; Greeff et al., 2012). Based on this finding, it is possible to assess whether an unknown RLK is involved in a biological process by recombining the NT of a known ligand RLK with the KD of the unknown RLK (Brutus et al., 2010).

Leveraging the Arabidopsis protoplasts PTI activation transient reporting system, the aforementioned recombinant RLK strategy (FLS2 NT + tested RLK KD) was employed to systematically screen 133 LRR-RLKs from the Arabidopsis genome. This comprehensive screen was designed to identify candidates that may play a role in the PTI pathway, and found 18.0% of the tested RLKs were able to complete PTI immune signaling and activate downstream reporter gene expression in Arabidopsis protoplasts after flg22 induction. In addition to identifying several RLKs that have been reported to participate in plant immune responses, such as At2g01950 (Wang et al., 2001), At1g17750 (Huffaker and Ryan, 2007), At5g01540 (LecRKA4.1) (Singh et al., 2012), At1g09970 (RLK7) (Hou et al., 2014), At2g13790 (de Oliveira et al., 2016), At4g28490 (Patharkar et al., 2017), and At5g65710 (HSL2) (Wang et al., 2017), it was also found that some RLKs that were not previously known to mediate PTI immune responses in protoplasts. It is well-established that proper subcellular localization is essential for protein function. RLKs act as plant receptor proteins situated on the plasma membrane, where they receive extracellular ligand signals and activate intracellular signaling pathways via their kinase domains (Hunter, 1995; Shiu and Bleecker, 2001). By constructing transgenic lines expressing 6 *rRLKs* (*FLS2 NT-At1g55610 KD*, *FLS2 NT-At2g37050 KD*, *FLS2 NT-At3g47570 KD*, *FLS2 NT-At2g28990 KD*, *FLS2 NT-At5g63930 KD*, *FLS2 NT-At2g01950 KD*) and conducting disease resistance characterization analysis, it was revealed that the recombination of RLK did not affect its correct subcellular localization, and further confirmed the involvement of these RLKs in plant disease resistance. These results indicate that the transient screening system has high credibility, and utilizing recombination strategy with the help of such system can promote our understanding of unknown RLK functions.

### 4.2 The identification of conserved amino acid sites within the KD domains facilitates the screening process for immune-related RLKs in plant genomes

Research on identified immune RLKs suggests that the patterns of recognition to pathogen invasion signal and the downstream signal transduction pathways triggered by different RLKs are remarkably similar (Bjornson et al., 2021; Huang et al., 2023). However, the reasons behind this commonality remain unclear. In this study, the NT domains of each RLK were artificially unified. That is, on the basis of excluding differences in ligand recognition or even recognition sensitivity, the same extracellular signal was transmitted into the cell through different KD domains. Although the degree of immune activation differed, there were still some commonalities. Through sequence analysis, it was discovered that certain conserved motifs exist within the identified immune-related RLKs, which are hypothesized to be part of the reason for the commonality in downstream immune responses mediated by different RLKs. Additionally, based on the standardized plant RLK bioinformatics analysis database (Yin et al., 2024), the discovery of these common features may assist researchers in the expeditious and efficacious identification of pertinent candidates involved in plant immunity among the numerous putative RLKs in the Arabidopsis genome, and even in other species. Once the target RLK is identified, its upstream specific ligand can be screened, its downstream immune-related interaction proteins can be identified, and the specific molecular mechanism of its involvement in PTI signaling pathway can be analyzed. The elucidation of the roles of these RLKs represents a considerable leap forward, establishing a foundation for deeper investigation into the processes governing plant immunity.

Studies to date have shown that expression of RLK can enhance the disease resistance of plants upon induction, while exhibiting no negative effects on the plant growth and development under normal growth conditions. The implication of this work could extend to identifying potential targets for improving crop resilience against pathogens by understanding and potentially enhancing the signaling pathways leading to immune responses.

## Data Availability

The data for the article is sourced from the Ensembl database, and the gene numbers of all RLKs mentioned in the article are listed in Supplementary Table S1. The DNA sequences can also be found on NCBI.

## Supplementary material

Supplementary Figure 1 Relative expression levels of *FRK1* when expressing 133 HBT-*FLS2 NT-RLK KD-GFP rRLKs* individually after flg22 treatment in Arabidopsis protoplasts. * indicates significant difference at P<0.05; ** indicates significant difference at P<0.01.

Supplementary Figure 2 Subcellular localization of rRLK-GFP proteins in transgenic plants. Confocal images of 6 rRLK-GFP fluorescence in root cells of plate-grown *fls2* transgenic plants expressing 6 rRLK-GFP individually. Bars = 50 μm.
