# Supplementary figures and images for: A large-scale screening identifies receptor-like kinases with common features in kinase domains that are potentially related to disease resistance in planta
Source: Front Plant Sci. 2024 Nov 13;15:1503773. doi: 10.3389/fpls.2024.1503773 (PMC11598347; doi:10.3389/fpls.2024.1503773)

Figure S1

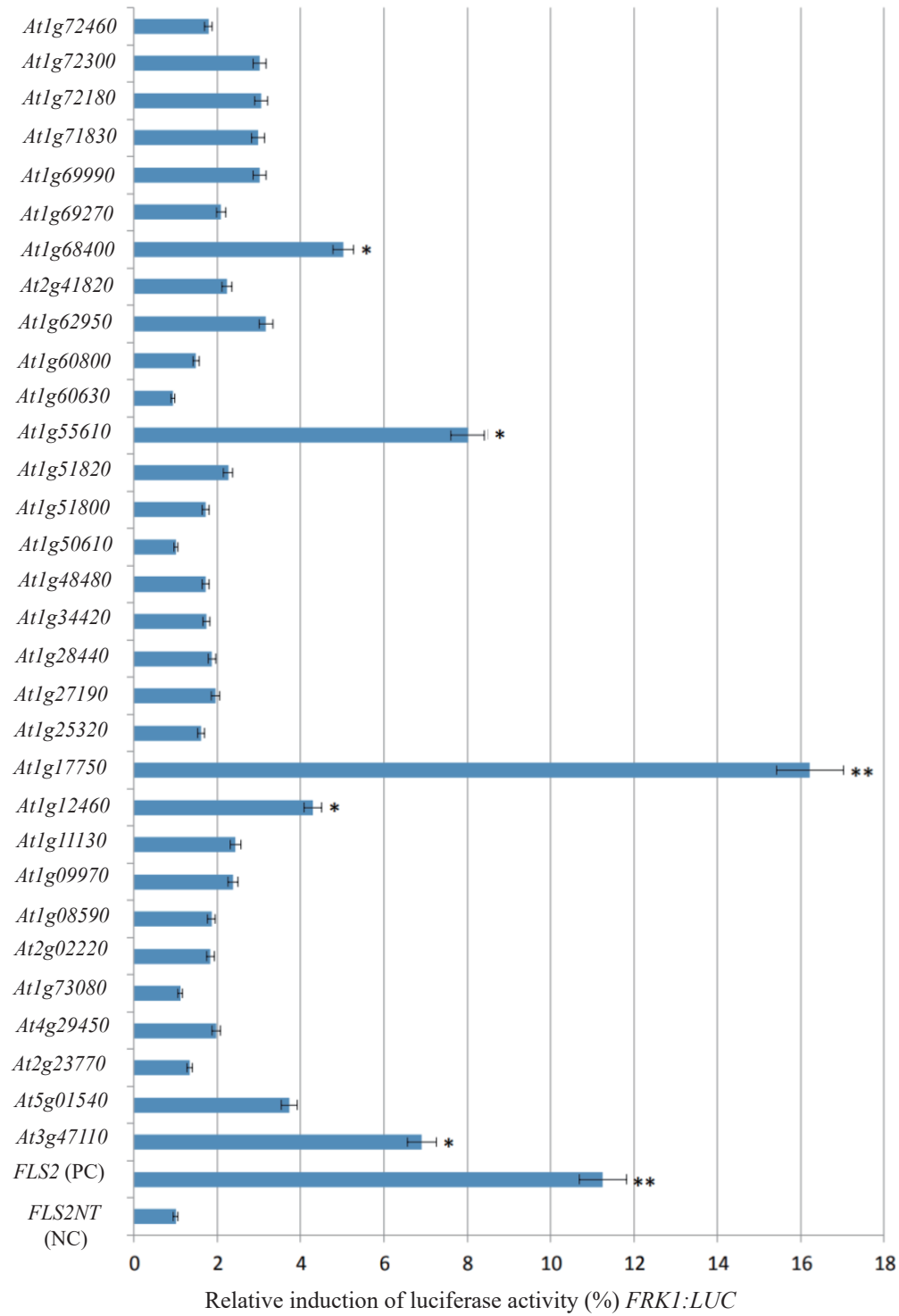

Figure S1

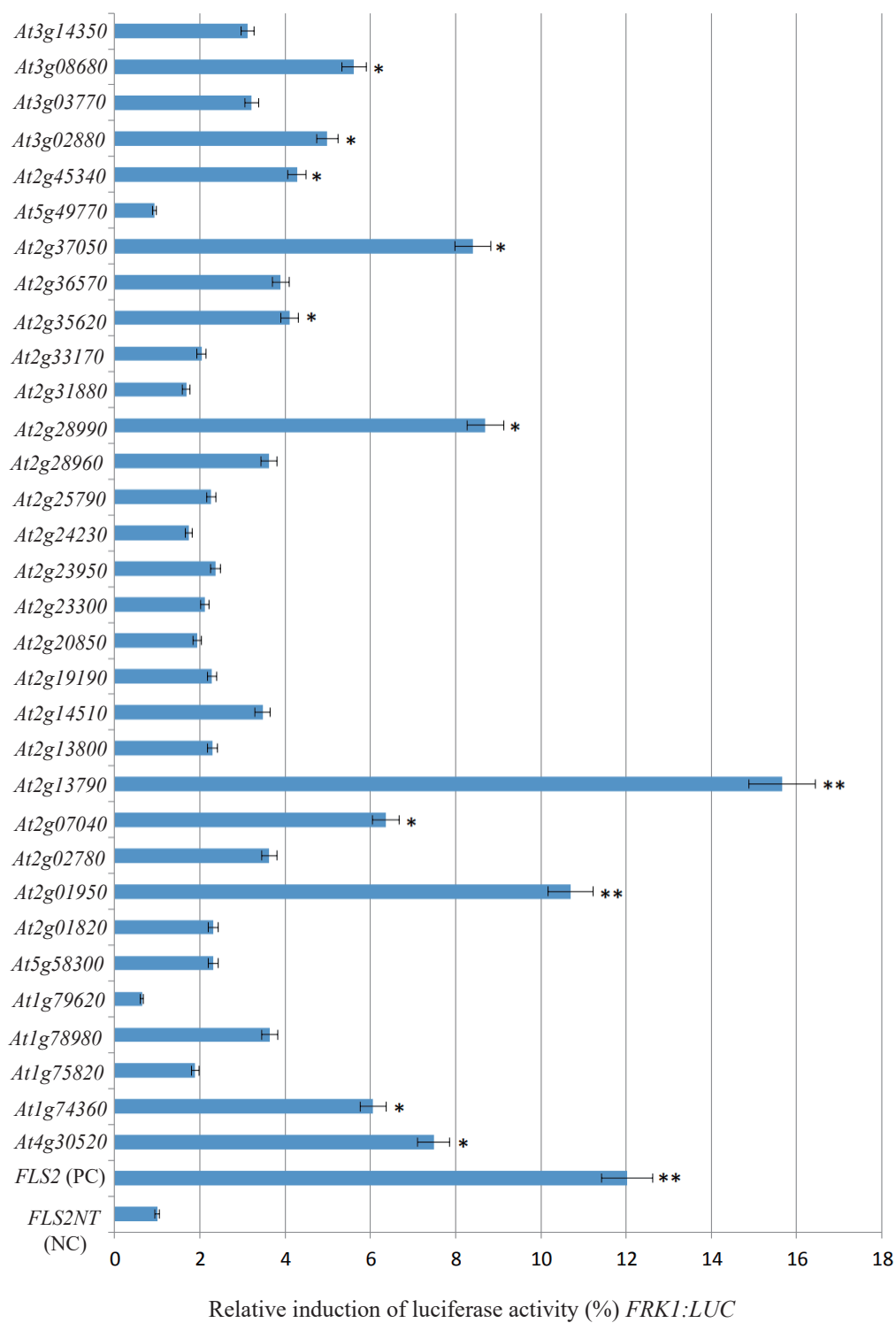

Figure S1

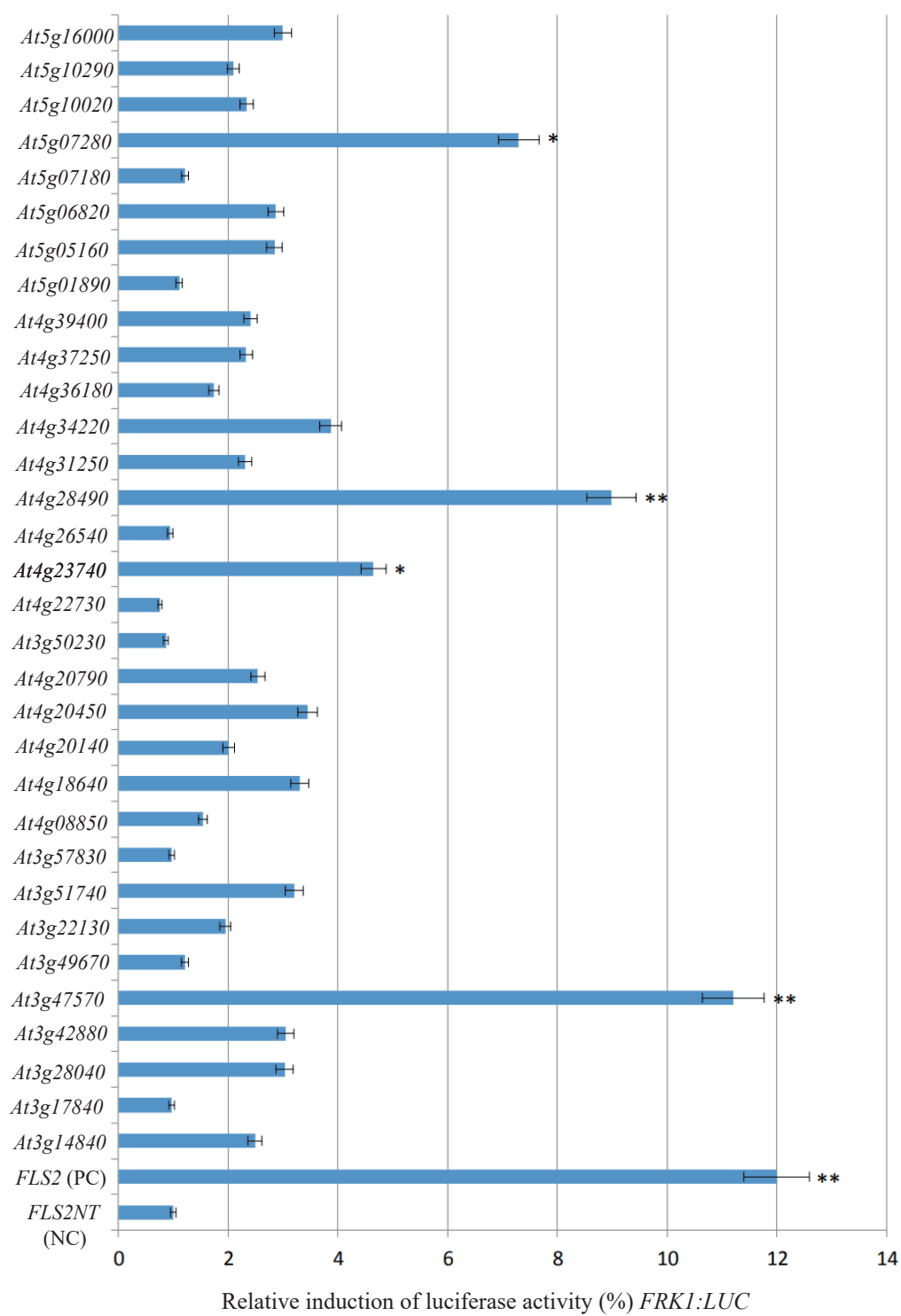

Figure S1

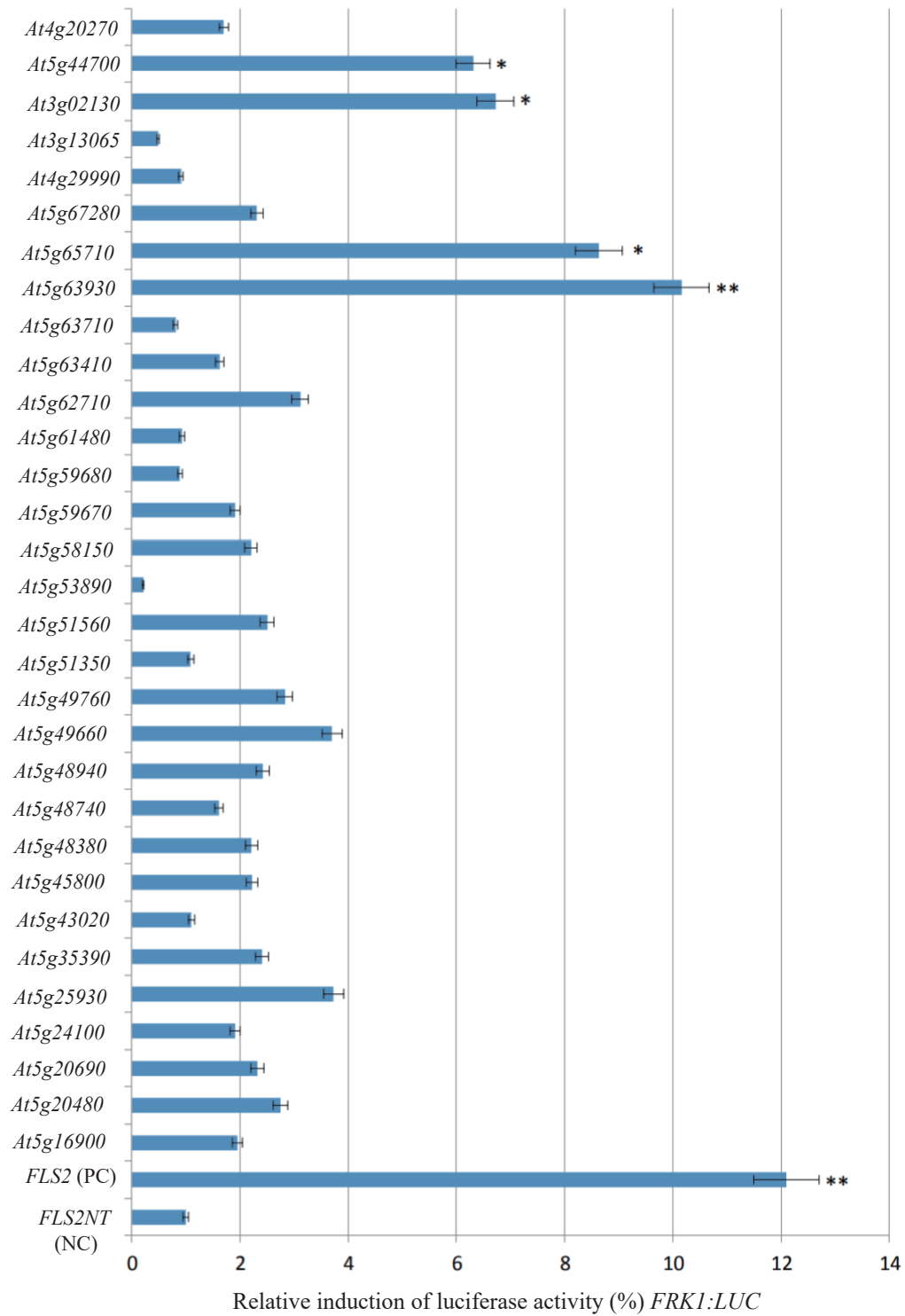

Figure S1

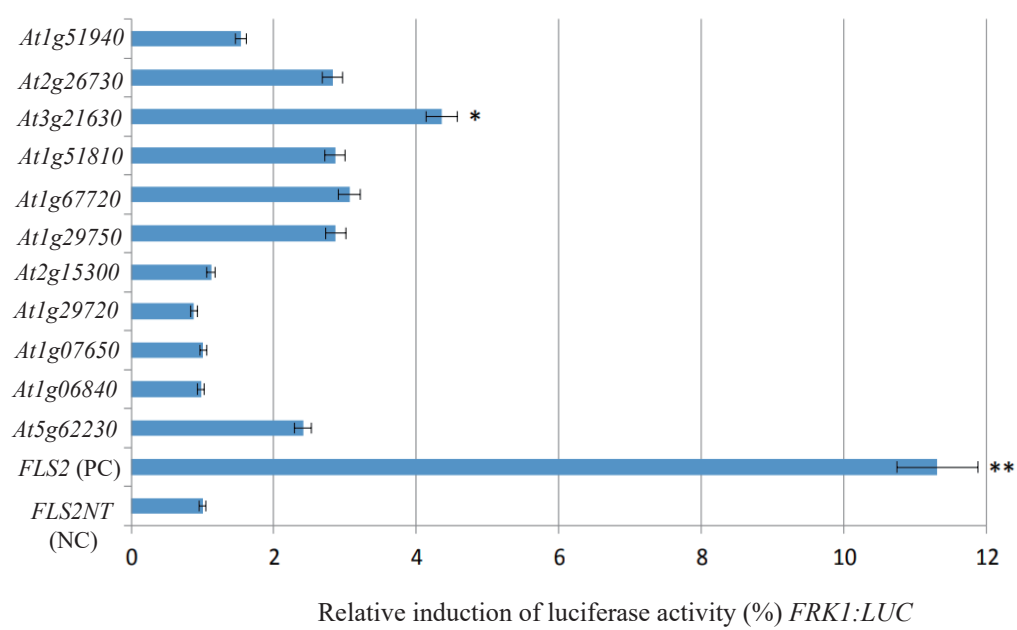

Supplement: Supplementary Figure 1 — Relative expression levels of FRK1 when expressing 133 HBT-FLS2 NT-RLK KD-GFP rRLKs individually after flg22 treatment in Arabidopsis protoplasts. * indicates significant difference at P<0.05; ** indicates significant difference at P<0.01. [file DataSheet1.pdf]

Figure S2

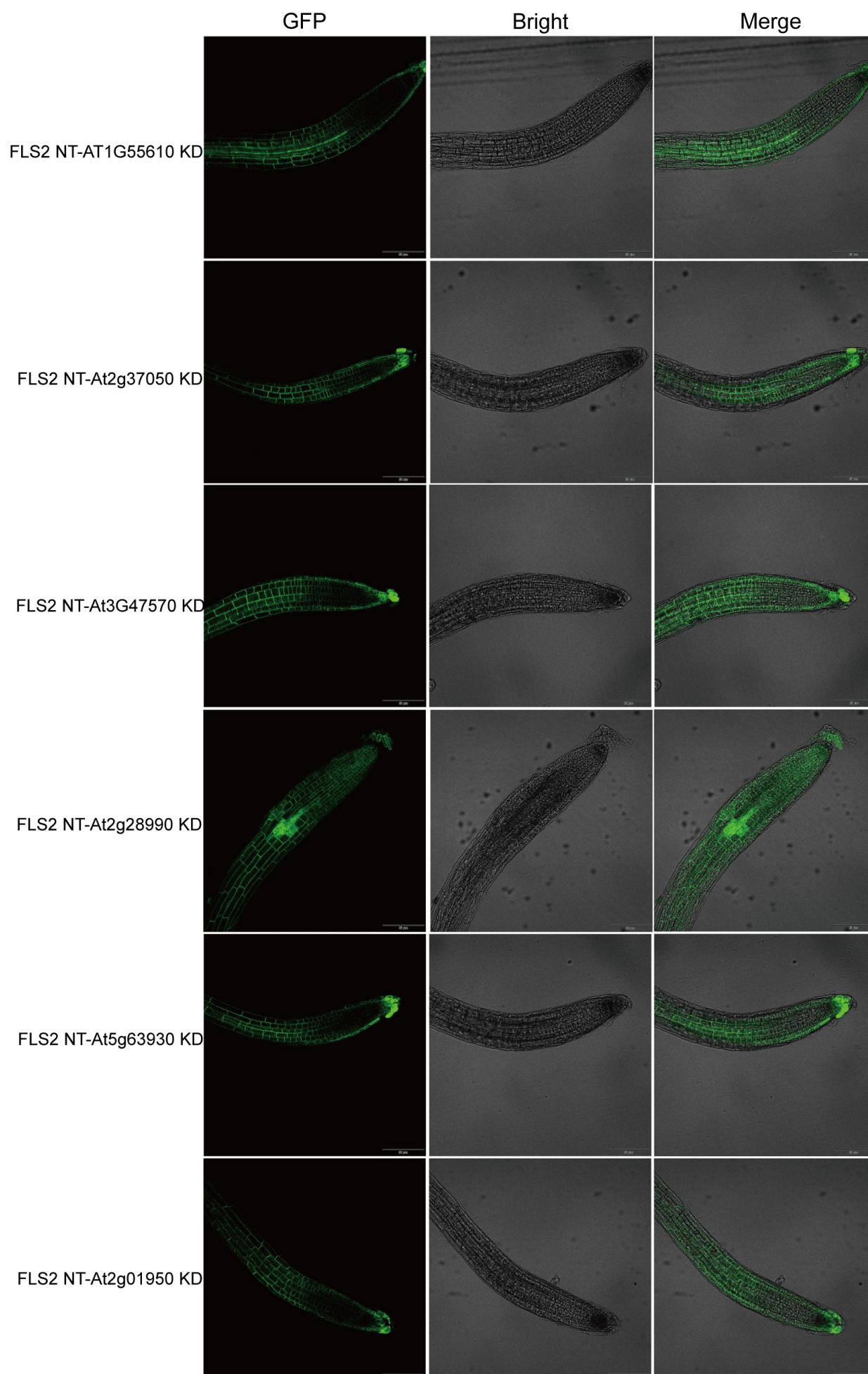

Supplement: Supplementary Figure 2 — Subcellular localization of rRLK-GFP proteins in transgenic plants. Confocal images of 6 rRLK-GFP fluorescence in root cells of plate-grown fls2 transgenic plants expressing 6 rRLK-GFP individually. Bars = 50 μm. [file DataSheet2.pdf]
